# Nodal Upstaging and Oncologic Outcomes After Segmentectomy Versus Lobectomy for Early-Stage Non-Small Cell Lung Cancer

**DOI:** 10.3390/cancers18061039

**Published:** 2026-03-23

**Authors:** Alecsandra Tudor, Ye Tian, Edoardo Zanfrini, Etienne Abdelnour-Berchtold, Jean Yannis Perentes, Thorsten Krueger, Michel Gonzalez

**Affiliations:** 1 Department of Thoracic Surgery, Lausanne University Hospital (CHUV), 1011 Lausanne, Switzerland; alecsandra-anca.tudor@chuv.ch (A.T.); ye.tian@unil.ch (Y.T.); edoardo.zanfrini@chuv.ch (E.Z.); etienne.abdelnour@chuv.ch (E.A.-B.); jean.perentes@chuv.ch (J.Y.P.); thorsten.krueger@chuv.ch (T.K.); 2 Faculty of Biology and Medicine, University of Lausanne (UNIL), 1011 Lausanne, Switzerland

**Keywords:** NSCLC, segmentectomy, lobectomy, recurrence, survival, lymph node dissection

## Abstract

Background: Segmentectomy is increasingly used and is emerging as a key treatment option for early-stage non-small cell lung cancer (NSCLC). However, questions remain regarding the adequacy of lymph node evaluation, particularly differences in N1 versus N2 dissection, and the implications for staging accuracy and adjuvant therapy. Methods: This narrative review synthesizes evidence from studies published between 2019 and 2025 comparing nodal upstaging, survival outcomes, and the role of completion lobectomy following segmentectomy versus lobectomy. Results: Twelve studies, including more than 175,000 patients, were analyzed. Lobectomy was associated with a significantly higher overall nodal upstaging rate (14.5% vs. 6.6%, *p* < 0.001), driven primarily by increased detection of N1 disease (13.3% vs. 3.7%, *p* < 0.001), while N2 upstaging rates were similar between procedures (5.5% vs. 3.2%, *p* = 0.07). Despite lower N1 detection, adjusted analyses reported comparable survival outcomes among patients with occult pathologic N1 (pN1) or N2 (pN2) disease who received adjuvant therapy. Segmentectomy provided outcomes comparable to lobectomy, whereas wedge resection was associated with inferior survival (HR 1.23, *p* = 0.042). Completion lobectomy has not demonstrated a consistent survival benefit and was associated with substantial morbidity in limited retrospective series, including high rates of thoracotomy conversion and major complications. Conclusions: When performed with systematic nodal dissection, adequate surgical margins, and appropriate adjuvant therapy, segmentectomy appears to provide survival outcomes comparable to lobectomy in selected patients with early-stage NSCLC. Completion lobectomy may not be routinely required and should be considered on a case-by-case basis within a multidisciplinary context. These findings support the use of segmentectomy in carefully selected patients when high-quality surgical staging and integrated oncologic care are ensured, while highlighting the need for prospective studies addressing occult nodal disease in the modern treatment era.

## Simple Summary

Segmentectomy is increasingly used to treat small, early-stage non-small cell lung cancer, as recent clinical trials have shown similar survival with better preservation of lung function. However, concerns remain that segmentectomy may detect fewer lymph node metastases, potentially leading to understaging and undertreatment. In this review, we examined contemporary studies comparing segmentectomy and lobectomy with a focus on nodal upstaging, survival outcomes, and the need for completion lobectomy when unexpected lymph node involvement is found. Across large national and multicenter studies, lobectomy identified nodal metastases more frequently than segmentectomy, mainly due to greater detection of lymph nodes located near the lung (N1 nodes). Importantly, survival and recurrence outcomes were similar between the two procedures once lymph node involvement was identified and appropriate postoperative treatments were given. These findings suggest that the lower rate of nodal upstaging after segmentectomy reflects differences in lymph node sampling rather than worse cancer control. When lymph nodes are carefully evaluated and modern adjuvant therapies are used, segmentectomy provides oncologic outcomes comparable to lobectomy, and routine completion lobectomy may not be required and should be considered on a case-by-case basis within a multidisciplinary setting.

## 1. Introduction

For nearly thirty years, lobectomy with systematic lymph node dissection was regarded as the gold standard for early-stage non-small cell lung cancer (NSCLC), due to evidence supporting superior local control compared to sub-lobar resection [1]. Recently, management of small, peripheral tumors has evolved following the pivotal JCOG0802/WJOG4607L and CALGB/Alliance 140,503 trials [2,3]. These multicenter randomized studies demonstrated that sub-lobar resection offers noninferior overall survival in patients with clinically node-negative tumors less than 2 cm in size. As a result, international guidelines increasingly include segmentectomy as a standard option for selected patients with early-stage NSCLC. These data primarily apply to small (≤2 cm), peripheral, clinically node-negative (cN0) tumors, and evidence remains more limited for larger, central, or high-risk lesions, including those with spread through air spaces (STAS) [4,5,6,7]. However, the oncologic adequacy of segmentectomy in cases with nodal involvement remains highly debated. The 2023 European Society of Thoracic Surgeons (ESTS) expert consensus states that lobectomy is preferred for patients with known pN1 or pN2 disease [8], although this recommendation is based largely on expert consensus and observational data, reflecting the limited availability of high-level evidence in this setting. Current consensus statements and retrospective series suggest adjuvant systemic therapy as the primary treatment for occult nodal disease, reserving completion lobectomy for cases requiring further margin or nodal assessment. Ongoing concerns persist regarding whether segmentectomy can achieve lymph node yields and tumor margins comparable to lobectomy, potentially affecting the detection of occult nodal metastasis and local control [9,10,11,12,13,14,15].

Large-scale analyses have identified differences in staging accuracy between lobectomy and segmentectomy. Lobectomy detects occult nodal metastases at higher rates than segmentectomy. Recent evidence suggests that this “upstaging gap” is primarily due to increased detection of N1 disease, while N2 detection rates are similar between procedures [9,10,12,14,15]. This likely reflects differences in intrapulmonary and peribronchial (N1) node retrieval rather than true biological differences. This distinction is clinically meaningful, as accurate staging determines eligibility for adjuvant therapy, which improves overall survival in node-positive patients regardless of surgical approach. The identification of occult nodal disease (pN+) creates a therapeutic dilemma: whether to perform a completion lobectomy to address potential residual disease or to rely solely on adjuvant systemic therapy. Historically, completion lobectomy was preferred for local control. However, recent studies have linked this approach to increased morbidity without a demonstrated survival benefit [16,17].

This narrative review synthesizes evidence published between 2019 and 2025. It examines the incidence of nodal upstaging and compares long-term survival outcomes in patients with occult node-positive disease treated with segmentectomy or lobectomy. Additionally, the review evaluates the utility and risks associated with completion lobectomy in current clinical practice.

## 2. Materials and Methods

### 2.1. Literature Search Strategy and Selection Criteria

A structured literature search was performed using PubMed/MEDLINE, Embase, Scopus, and the Cochrane Library, including studies published from January 2019 to November 2025 to reflect contemporary surgical practice and modern oncologic management. The search strategy combined predefined keywords and Medical Subject Headings (MeSH) terms using Boolean operators (AND, OR), including ‘non-small cell lung cancer’, ‘segmentectomy’, ‘lobectomy’, ‘nodal upstaging’, ‘lymph node’, and ‘survival’. Additionally, the references from the included articles were manually reviewed to identify further relevant studies. Only peer-reviewed articles published in English were included. Conference abstracts, preprints, and non–peer-reviewed sources were excluded.

### 2.2. Inclusion and Exclusion Criteria

Studies were included if they met the following criteria: (1) adult patients with tumor size equal to or smaller than 2 cm, who underwent segmentectomy or lobectomy; (2) direct comparison of nodal upstaging rates (cN0 to pN1/N2) and/or survival outcomes between the two procedures; and (3) availability of full-text data in English.

The review focused on anatomical segmentectomy and lobectomy. Studies including wedge resections were excluded unless outcomes for segmentectomy were reported separately. For mixed ‘sublobar’ cohorts, studies were included only when segmentectomy-specific data were available or when segmentectomy constituted the predominant surgical approach. Studies focusing exclusively on wedge resections were excluded, as these non-anatomic procedures differ oncologically from segmentectomy, particularly with regard to lymph node retrieval and local control. Included studies primarily involved clinically node-negative (cN0 or cStage IA) patients, based on standard preoperative staging modalities including computed tomography (CT), positron emission tomography (PET-CT), and, when available, endobronchial ultrasound (EBUS), as reported in the individual studies.

The review concentrated on upfront surgical resection; therefore, studies including neoadjuvant therapy were excluded when this constituted a substantial proportion of the cohort. However, large database analyses including a small proportion of patients receiving induction therapy were retained. This potential source of confounding was considered in the interpretation of results.

### 2.3. Data Extraction and Quality Assessment

Data extraction, using the PICOS framework [18], targeted three primary domains: (1) the prevalence and distribution of occult nodal disease, (2) survival outcomes, and (3) the role of completion lobectomy. Nodal upstaging was analyzed by stratifying cases into N1 (hilar/peribronchial) and N2 (mediastinal) disease. Data were collected on the number of lymph nodes resected, the stations sampled, and the differentiation between intrapulmonary/peribronchial N1 nodes from lung specimens and those dissected from the hilum. Oncologic outcomes, including overall survival (OS) and recurrence-free survival (RFS), were summarized. Because we are aware of the selection bias inherent in retrospective data—where lobectomy is often selected for larger or more metabolically active tumors—we actively sought studies reporting statistical adjustments, such as propensity score matching and overlap weighting, as we identified these methods to be the best available for balancing baseline characteristics. Data on adjuvant systemic therapy were extracted when available; however, the specific type of therapy (chemotherapy, immunotherapy, or targeted therapy) was inconsistently reported across studies.

Finally, data on the morbidity and survival impact of completion lobectomy for unexpected nodal metastasis were extracted, with particular attention to conversion rates to thoracotomy and perioperative complications.

The selected publications encompassed multi-institutional cohorts, national database analyses, and single-center experiences. Detailed single- and multicenter studies contributed with more granular data, often absent from national registries, such as recurrence patterns (locoregional versus distant), the impact of SUVmax on upstaging risk, and adherence to lymph node sampling guidelines. The integration of these diverse data sources allowed a more nuanced assessment of whether the lower upstaging rates observed with segmentectomy are attributable to differences in sampling, and whether they are associated with inferior OS or RFS. In addition, the methodological quality of the included observational studies was assessed using the Newcastle–Ottawa Scale (NOS), which evaluates studies based on three domains: selection of study groups, comparability of cohorts, and assessment of outcomes. Each study was independently reviewed, and scores were assigned to provide an overall assessment of risk of bias. The results of this quality assessment are summarized in Supplementary Table S1. No quantitative meta-analysis was performed, and no pooled estimates (hazard ratios or odds ratios) were calculated. Results are presented as a descriptive synthesis of the available evidence.

## 3. Results

A total of 12 publications, ranging from 1 January 2019 to 1 November 2025, were included, from large national database analyses and multicenter registries to institutional series. Sample sizes varied widely, from small cohorts to over 170,000 patients. These studies either directly compared segmentectomy and lobectomy outcomes in patients with nodal-positive disease (pN1 and/or pN2) or provided single-arm segmentectomy data with relevant oncologic and nodal information [2,3,9,10,11,12,13,14,15,16,19,20,21,22,23,24,25]. Follow-up durations across publications ranged from 24 to 74 months. Notably, heterogeneity exists among the included studies, including differences in study design (single-center versus large national databases), geographic populations (Asian versus Western cohorts), surgical approach (minimally invasive versus open surgery), and the extent of mediastinal lymph node dissection, which may contribute to variability in reported upstaging rates and survival outcomes.

### 3.1. N1 Disease

The incidence of occult N1 disease was consistently lower after segmentectomy compared to lobectomy across studies, with this difference appearing related to the extent of lymph node retrieval (Table 1). In studies reporting nodal counts, fewer intrapulmonary and hilar lymph nodes were resected during segmentectomy than during lobectomy. Despite these differences in N1 detection, adjusted survival analyses among patients with occult pN1 disease showed no statistically significant difference in overall survival between segmentectomy and lobectomy. Similar findings were reported in large database analyses and propensity-adjusted cohorts, with comparable long-term survival among patients with occult pN1 disease who received adjuvant therapy.

### 3.2. N2 Disease

In contrast to N1 findings, rates of occult N2 upstaging were generally similar between segmentectomy and lobectomy, particularly in studies reporting routine mediastinal lymph node dissection (Table 2). Among patients with occult pN2 disease, adjusted analyses consistently demonstrated no statistically significant difference in overall survival between surgical approaches. These findings were observed across both large database studies and multicenter cohorts, although results should be interpreted in the context of retrospective study design and heterogeneity in staging practices.

### 3.3. Overall Nodal-Positive (N+) Cohorts

Across studies including combined occult N1 and N2 disease, segmentectomy demonstrated survival outcomes comparable to lobectomy in adjusted analyses (Table 3). These findings were consistent across large database studies and propensity-matched cohorts, with no statistically significant differences in overall or recurrence-free survival between procedures.

In contrast, wedge resection was associated with inferior survival outcomes and should not be considered equivalent to anatomical segmentectomy. Importantly, this negative signal should not be extrapolated to segmentectomy, which consistently demonstrated outcomes comparable to lobectomy when adequate staging and adjuvant therapy were applied.

Although some studies reported higher local recurrence rates following segmentectomy, this did not translate into worse survival outcomes in adjusted analyses.

### 3.4. Predictors of Occult Nodal Disease

Risk factors for occult nodal metastasis were consistent across studies. Huang et al. [24] identified high PET SUVmax as the only independent predictor of nodal upstaging, with an odds ratio of 3.50 for tumors with SUVmax > 4.5. Razi et al. [15] found that squamous histology and larger tumor size were significant predictors, while Jacobs et al. [9] also identified tumor size and specific histologic subtypes (other than adenocarcinoma) as significant predictors of upstaging.

### 3.5. Lymph Node Yield

Segmentectomy was generally associated with lower lymph node yields compared to lobectomy, reflecting differences in the extent of nodal dissection. Suzuki et al. [10] found that segmentectomy resulted in fewer lymph nodes resected than lobectomy (median 12 versus 18; *p* < 0.001). Luo et al. [19] reported similar findings but noted that survival was not compromised by lower lymph node yields when adjuvant therapy was administered. Adjuvant therapy emerged as the strongest predictor of survival in pN+ patients, improving outcomes regardless of resection type [15] (HR 0.61 for pN1 and 0.68 for pN2; *p* < 0.01). However, reduced lymph node retrieval did not appear to adversely impact survival outcomes when appropriate adjuvant therapy was administered.

### 3.6. Recurrence Patterns

Although overall survival was similar between procedures, distinct recurrence patterns were observed. In a multicenter retrospective analysis, local recurrence rates were significantly higher following segmentectomy compared to lobectomy (35.5% vs. 21.4%; *p* = 0.013), with recurrences occurring primarily at the staple line and within the preserved lobe [11]. Nevertheless, distant recurrence remained the predominant failure pattern across all studies, occurring in up to 70% of relapsed cases [20].

### 3.7. Role of Completion Lobectomy

Recent evidence questions the therapeutic benefit of completion lobectomy for occult nodal disease. Luo et al. [19], in a large analysis including over 120,000 patients, found no survival advantage associated with completion lobectomy in pN2 disease, while any early survival benefit observed in pN1 disease diminished over time. Similarly, Bongiolatti et al. [16] reported substantial perioperative morbidity associated with re-operations, with 74% of procedures requiring conversion to thoracotomy due to dense hilar adhesions and 22% of patients experiencing major complications (Clavien-Dindo > II), despite the absence of operative mortality.

These findings are consistent with data from recent multi-institutional series, in which completion lobectomy is associated with high technical complexity, including frequent adhesions (reported in up to 94–100% of cases), conversion rates ranging from 0% to 20%, pulmonary artery injury rates of approximately 11–30%, and postoperative complication rates ranging from 10% to 55% [17].

Nevertheless, more recent multi-institutional data suggest that completion lobectomy can be performed with acceptable perioperative outcomes and no perioperative mortality in selected patients, achieving 5-year overall survival rates of approximately 58% in cases of local recurrence [17]. However, these findings are based on small retrospective cohorts and should be interpreted with caution.

## 4. Discussion

With this review, we wish to illuminate a critical paradox in the surgical management of early-stage non-small cell lung cancer (NSCLC): although lobectomy is more effective at detecting occult N1 nodal metastases than segmentectomy, this diagnostic advantage has not been associated with a clear overall survival benefit in the available retrospective cohorts. Across these cohorts totaling over 170,000 patients, long-term survival for occult pN1 and pN2 disease appeared comparable between procedures in adjusted retrospective analyses when adjuvant systemic therapy was administered. These findings question the traditional view that completion lobectomy is mandatory and suggest that it may not be routinely required in all cases. Importantly, the available evidence is derived predominantly from retrospective, adjusted analyses and should therefore be interpreted with caution when inferring equivalence between surgical strategies.

The “upstaging gap” between procedures is well-documented. Large-scale analyses consistently demonstrate that lobectomy yields higher overall nodal upstaging (10–15%) compared to segmentectomy (3–7%). However, the data from Suzuki et al. [10] reveal that this disparity is driven almost exclusively by the retrieval of peribronchial N1 lymph nodes. In their institutional series, N1 upstaging was nearly fourfold higher in lobectomy (13.3% vs. 3.7%; *p* < 0.001), likely due to the removal of intrapulmonary nodes that remain embedded in the preserved segments during sublobar resection. These ranges should be interpreted in the context of heterogeneous study populations, including large database analyses and single-center series from both Eastern and Western cohorts [26,27,28].

Importantly, this disparity does not extend to mediastinal nodes. Upstaging to N2 disease was statistically similar between lobectomy and segmentectomy across multiple studies (Suzuki: 5.5% vs. 3.2%; Razi: 3.9% vs. 2.4%) [10,15]. These findings suggest that when guideline-concordant lymphadenectomy is performed, segmentectomy provides mediastinal staging accuracy equivalent to that of lobectomy.

Despite the lower rate of N1 detection, survival outcomes for upstaged patients were remarkably similar. In the largest propensity-matched analysis to date, Jacobs et al. found no difference in overall survival for patients with occult pN1 disease treated by segmentectomy versus lobectomy (HR 1.04; 95% CI 0.65–1.66) [9]. Similarly, Razi et al. [15] reported identical 5-year survival rates for occult pN2 disease (41.6% vs. 37.2%; *p* > 0.05). These findings are supported by Liou et al. [13], who analyzed over 2400 patients with occult N2 disease and found no difference in survival between sublobar resection and lobectomy (HR 0.97; *p* = 0.79). While survival outcomes were equivalent between segmentectomy and lobectomy, differences in recurrence patterns warrant careful consideration. Several studies reported higher local recurrence rates following segmentectomy, particularly at the staple line or within the preserved lobe [29,30,31]. Importantly, local recurrence represents a clinically meaningful endpoint beyond overall survival. Recurrences frequently require additional interventions such as stereotactic body radiotherapy (SBRT), repeat surgical resection, or systemic therapy, each of which may be associated with morbidity, impact on pulmonary function, and increased healthcare costs [31]. While many of these treatments are effective in achieving local control, they may compromise quality of life and expose patients to cumulative treatment-related toxicity. Therefore, the burden of recurrence should be carefully considered when interpreting the apparent equivalence in survival outcomes between segmentectomy and lobectomy.

A notable exception is the study by Iwai et al. [21], which reported a survival advantage for lobectomy in T1N2 patients (HR 0.96; *p* = 0.001). However, this result should be interpreted in light of cohort selection, as this cohort included a significant proportion of patients (11.5%) who received neoadjuvant chemotherapy, indicating the presence of known (clinical) N2 disease. For these patients, lobectomy remains the standard of care. In contrast, for cN0 patients with truly occult N1 status, the evidence supports oncologic equivalence between segmentectomy and lobectomy: Liou et al. [13] found no survival deficit with sublobar resection in occult N2 disease, and Mynard et al. [14] confirmed survival equivalence between segmentectomy and lobectomy for N+ disease, with wedge resection the only factor independently associated with inferior outcomes. With the increasing use of adjuvant immunotherapy and targeted therapies in node-positive disease, accurate nodal staging is likely to become even more relevant than the extent of parenchymal resection.

It is imperative to distinguish segmentectomy from wedge resection when discussing occult nodal disease. Mynard et al. [14] demonstrated that while segmentectomy and lobectomy offered equivalent median survival for occult N+ disease (68.5 vs. 57.6 months; *p* = 0.20), wedge resection was independently associated with worse overall survival (HR 1.23; *p* = 0.042). This distinction likely reflects the non-anatomic nature of wedge resection, which fails to remove the tumor’s specific lymphatic drainage basin (N1), confirming that segmentectomy is the minimum oncologically effective sublobar resection for node-positive cases.

The identification of occult nodal disease frequently prompts consideration of completion lobectomy. However, recent biological and clinical evidence strongly challenge the therapeutic value of this approach. Biologically, the rationale that completion lobectomy clears “residual” local disease is contradicted by pathologic evidence. Nomori et al. [20] analyzed completion lobectomy specimens from ten patients with occult N1/N2 disease and found no residual malignancy in the remaining lobe or its lymph nodes in any case. Furthermore, in patients who did not undergo complete resection, local recurrence was absent, with failures occurring exclusively at distant sites. This suggests that segmentectomy with systematic lymph node dissection provides sufficient local control, and extending the resection offers no additional oncologic yield.

Clinically, Luo et al. [19] conducted a time-dependent analysis demonstrating no survival benefit for completion of a lobectomy. For occult pN2 disease, outcomes were identical between procedures. For occult pN1 disease, segmentectomy showed a survival advantage during the first two years (HR 0.67; *p* = 0.03), which was attributable to preserved lung function and reduced surgical trauma, before outcomes converged with those of lobectomy. These findings suggest that immediate conversion to lobectomy eliminates the early perioperative benefits of segmentectomy without providing any long-term oncologic advantage.

From a technical perspective, completion lobectomy is associated with considerable morbidity [16,17]. In the era of patient-centered care, postoperative quality of life and the ability to receive timely adjuvant therapy are increasingly important considerations. 0% mortality is no longer an end in itself. Increasingly, the projected quality of life after surgery is becoming an ever more important factor in the decision-making process. Weighing heavily in this projection is the need for adjuvant therapy, a rapid postoperative recovery, and patient age. Given the absence of a survival benefit and the substantial risk of surgical morbidity, routine completion lobectomy for occult N+ disease is not justified. Taken together, these findings support a risk-adapted surgical strategy in which segmentectomy, combined with systematic nodal evaluation and appropriate adjuvant therapy, represents a valid option for selected early-stage NSCLC patients.

## 5. Limitations

This review is subject to several limitations inherent to the included literature.

Firstly, the majority of data were derived from retrospective observational studies, including large national registries such as the NCDB [21,22] and single-institution series. While propensity score matching and overlap weighting were employed in key studies to mitigate selection bias, unmeasured confounders, such as patient frailty, specific pulmonary function parameters, and surgeon preference, may still influence the choice of procedure and survival outcomes. Secondly, the definition of “occult” nodal disease varied across studies based on the rigor of preoperative staging. The usage of invasive mediastinal staging (EBUS/mediastinoscopy) and PET/CT was not uniform, potentially affecting the baseline probability of detecting nodal metastases. Secondly, large databases often lack granular data on the specific method of lymph node assessment (sampling vs. systematic dissection), resection margin, and recurrence patterns (local vs. distant), which limits the ability to directly correlate surgical technique with locoregional failure. Thirdly, while this review focuses on occult disease, the inclusion of a minority of patients receiving neoadjuvant therapy in certain cohorts (e.g., Iwai et al. [21]) introduces heterogeneity regarding the true “occult” nature of N2 disease. Fourthly, the patients included in the publications received staging and treatment as far back as 2002, stage definition as per the TNM classification, as well as (neo-)adjuvant therapy regimens, vary accordingly.

Finally, the long-term survival data for segmentectomy in the specific setting of N+ disease are still maturing compared to the decades of data available for lobectomy, and the impact of modern adjuvant regimens (immunotherapy and targeted therapy) on this specific surgical subpopulation remains to be fully defined. Although the included studies were published between 2019 and 2025, several large database analyses included patients treated as early as 2004. Therefore, a substantial proportion of the data reflects a pre-immunotherapy era, during which adjuvant treatment consisted primarily of platinum-based chemotherapy. As a result, the applicability of these findings to patients treated with modern immunotherapy or targeted therapies should be interpreted with caution. Furthermore, detailed information regarding the type of adjuvant systemic therapy (chemotherapy versus immunotherapy or targeted therapy) was not consistently available across studies, particularly in large national databases. This limitation precluded subgroup analyses based on treatment modality, despite the well-established impact of systemic therapy on survival in node-positive disease.

### Clinical Implications and Future Directions

The findings of this review have direct implications for thoracic surgical practice. The observed equivalence in survival outcomes between segmentectomy and lobectomy for occult pN1/N2 disease warrants, in our view, that the traditional mandate for completion lobectomy should be reconsidered. In the current era, when adjuvant systemic therapy largely determines prognosis in node-positive early-stage NSCLC, the biological benefit of removing additional lung parenchyma is minimal compared with the associated risks. Therefore, given the high morbidity and conversion rates to thoracotomy with completion lobectomy, this procedure may be best reserved for patients with positive parenchymal margins or other clear indications for further resection, rather than performed solely for occult nodal disease in otherwise adequately resected cases. With the increasing use of adjuvant immunotherapy and targeted therapies in node-positive disease, accurate nodal staging is likely to become even more relevant than the extent of parenchymal resection. However, such strategies require validation in prospective, ideally randomized or well-designed pragmatic trials before any definitive conclusions regarding superiority or equivalence can be drawn.

It is essential for clinicians to distinguish between anatomic segmentectomy and wedge resection, as wedge resection remains oncologically inferior for occult nodal disease and should be avoided in patients eligible for anatomic resection. Additionally, the ‘upstaging gap’ underscores the importance of systematic and standardized intraoperative lymph node dissection during segmentectomy. In practical terms, adequate nodal evaluation should include retrieval of segment-specific N1 (hilar and intrapulmonary) nodes and sampling of multiple mediastinal (N2) stations—typically at least three stations—as recommended by international guidelines. Given the variability in reported lymph node counts and their dependence on anatomical and pathological factors, emphasis should be placed on the anatomical completeness of nodal assessment rather than on a fixed numerical threshold. Particular attention should be given to the dissection of intrapulmonary and peribronchial (N1) nodes, which are more likely to be missed during segmentectomy, to ensure accurate staging and facilitate access to adjuvant therapies. Collectively, these limitations indicate that our findings should be interpreted as hypothesis-generating and supportive of cautious de-escalation in selected patients, rather than as definitive practice-changing evidence.

Future research should prioritize integrating molecular staging and advanced imaging techniques to improve preoperative prediction of occult nodal involvement. High SUVmax has been identified as a strong predictor of upstaging, indicating that patients with hypermetabolic tumors may benefit from more aggressive preoperative invasive staging. As indications for adjuvant immunotherapy and targeted agents expand, future trials should specifically assess whether the “segmentectomy plus adjuvant therapy” approach provides superior quality of life and equivalent survival compared to lobectomy in prospective, biomarker-driven cohorts.

## 6. Conclusions

The management of early-stage NSCLC is evolving, and the identification of occult nodal disease following segmentectomy presents a complex clinical challenge. This review aims to highlight that although lobectomy is associated with higher rates of nodal upstaging, particularly in N1 disease, this diagnostic advantage has not been associated with a clear survival benefit in available retrospective analyses for patients with occult pN1 or pN2 disease. When adjuvant systemic therapy is administered, long-term survival outcomes are equivalent between segmentectomy and lobectomy, indicating that prognosis is more strongly influenced by systemic disease control than by the extent of local resection.

Moreover, current evidence does not support our understanding of the routine use of completion lobectomy for occult nodal disease. Recent data show that completion lobectomy provides no survival advantage over segmentectomy for pN+ disease and is associated with significant morbidity, including high rates of conversion to thoracotomy and major perioperative complications. Therefore, for patients with clinical stage IA NSCLC and tumor size ≤ 2 cm who undergo anatomic segmentectomy and are found to have occult nodal metastases, we suggest that adjuvant systemic therapy may be favored over re-resection in this context, although decisions should remain individualized within a multidisciplinary setting.

## Figures and Tables

**Table 1:** N1 Upstaging (cN0 to pN1) After VATS Segmentectomy and Lobectomy.

Study	Design	Population	Resection Type	Surgical Approach	LN Dissection	N1 Upstaging (%)	*p*-Value	OS in pN1	*p*-Value
Suzuki 2025 [10]	Single-center	cT1N0M0 NSCLC	Segmentectomy vs. lobectomy	Predominantly VATS/RATS (>90%)	Station-based analysis; no uniform mandated protocol	3.7 vs. 13.3	<0.001	Comparable OS	Not reported (*p*-value not specified)
Razi 2020 [15]	NCDB	cT1N0M0 NSCLC	Segmentectomy vs. lobectomy	Not reported (NCDB; not stratified by group)	Not reported (extent of mediastinal dissection not available)	2.5 vs. 6.7	Not reported (N1-specific *p*-value not provided)	41.9% vs. 44.3%	0.35
Jacobs 2025 [9]	NCDB	cT1cN0M0 NSCLC	Segmentectomy vs. lobectomy (wedge excluded)	Mixed; not reported separately by procedure	LN count required; exact dissection type not reported	6.6 vs. 14.5 (pN+)	Not reported (N1 not separated)	HR 1.04 (95% CI 0.65–1.66)	Not significant
Mynard 2022 [14]	NCDB	cStage IA NSCLC	Segmentectomy vs. lobectomy (wedge analyzed separately)	Not reported (NCDB)	Not reported	2.0 vs. 4.7	Not reported (N1-specific *p*-value not provided)	68.5 vs. 57.6 months	0.20
Abdallat et al. 2022 [12]	Multicenter	Early-stage NSCLC	Sublobar (segmentectomy ± wedge) vs. lobectomy	Not reported	Not reported	Not reported (N1 rate not specified separately)	—	68.4% vs. 57.2% (occult vs. known N1)	Not reported

**Table 2:** N2 Upstaging (cN0 to pN2) After VATS Segmentectomy and Lobectomy.

Study	Design	Population	Resection Type	Surgical Approach	LN Dissection	N2 Upstaging (%)	*p*-Value	OS in pN2	*p*-Value
Suzuki 2025 [10]	Single-center	cT1N0M0 NSCLC	Anatomic segmentectomy vs. lobectomy	Predominantly VATS/RATS (>90%)	Station-based analysis; no uniform mandated protocol	3.2 vs. 5.5	0.074	Comparable OS	Not reported (*p*-value not specified)
Razi 2020 [15]	NCDB	cT1N0M0 NSCLC	Segmentectomy vs. lobectomy	Not reported (NCDB; not stratified)	Not reported (extent of mediastinal dissection not available)	2.4 vs. 3.9	Not reported (N2-specific *p*-value not provided)	41.6% vs. 37.2%	0.99
Luo 2024 [19]	NCDB	cStage IA NSCLC	Segmentectomy vs. lobectomy	Mixed (open, VATS, robotic; not stratified)	Not reported (minimum LN threshold used in sensitivity analyses)	1.9 vs. 3.7	Not reported (N2-specific *p*-value not provided)	HR 0.96	0.70
Liou 2022 [13]	NCDB	Stage IA NSCLC with pN2	Sublobar (segmentectomy ± wedge) vs. lobectomy	Not reported	Not reported	Not applicable (pN2-only cohort)	—	46.6% vs. 45.2%	0.319
Mynard 2022 [14]	NCDB	cStage IA NSCLC	Segmentectomy vs. lobectomy (wedge analyzed separately)	Not reported	Not reported	1.8 vs. 3.0	Not reported (N2-specific *p*-value not provided)	Not reported (pN2 not analyzed separately)	—

**Table 3:** Overall N+ Upstaging (cN0 to pN+) After VATS Segmentectomy and Lobectomy.

Study	Design	Population	Resection Type	Surgical Approach	LN Dissection	OS (Seg vs. Lob)	*p*-Value	Key Findings	
Jacobs 2025 [9]	NCDB	cT1cN0M0 NSCLC with pN+	Segmentectomy vs. lobectomy (wedge excluded)	Mixed; not stratified	LN count required; exact dissection type not reported	HR 1.04 (95% CI 0.65–1.66)	Not significant	Comparable OS after propensity matching	
Mynard 2022 [14]	NCDB	cStage IA NSCLC with occult N+	Segmentectomy vs. lobectomy (wedge analyzed separately)	Not reported	Not reported	68.5 vs. 57.6 months	0.20	No OS difference; wedge inferior (HR 1.23, *p* = 0.042)	
Ryuko 2025 [11]	Multicenter	cN0 NSCLC with pN1–N2	Segmentectomy vs. lobectomy	Mixed (institution-dependent)	Not standardized	OS comparable after adjustment	Not significant	Higher local recurrence with segmentectomy	
Nobel 2024 [20]	Single-center	cT1N0M0 NSCLC with pN+	Segmentectomy vs. lobectomy	Mixed (more open in lobectomy group)	Institutional practice	63% vs. 50% (5-year OS)	0.60	No difference in OS or recurrence	
Razi 2020 [15]	NCDB	cT1N0M0 NSCLC with pN1–N2	Segmentectomy vs. lobectomy	Not reported		Not reported	N1: 41.9% vs. 44.3%	0.35	No OS difference
							N2: 41.6% vs. 37.2%	0.99	Same for pN2 subgroup
Suzuki 2025 [10]	Single-center	cT1N0M0 NSCLC	Segmentectomy vs. lobectomy	Predominantly VATS/RATS		Station-based analysis	Median OS 103.6 vs. 106.3 months	0.576	No OS difference despite lower LN yield

## Data Availability

No new data were created or analyzed in this study. Data sharing is not applicable to this article.

## Supplementary Materials

The following supporting information can be downloaded at https://www.mdpi.com/article/10.3390/cancers18061039/s1. Table S1: Quality Assessment of Included Studies Using the Newcastle–Ottawa Scale (NOS).

## Abbreviations

NSCLC	Non-small cell lung cancer
HR	Hazard ratio
JCOG	Japan Clinical Oncology Group
CALGB	Cancer and Leukemia Group B
ESTS	European Society of Thoracic Surgeons
OS	Overall Survival
RFS	Recurrence Free Survival
cN0	clinically node-negative
pN1	pathologic N1
pN2	pathologic N2
pN+	pathologic node-positive
VATS	video-assisted thoracoscopic surgery
NCDB	National Cancer Database
UPMC	University of Pittsburgh Medical Center
MSKCC	Memorial Sloan Kettering Cancer Center
PSM	propensity score matching
MV Cox	multivariable Cox regression
SUVmax	maximum standardized uptake value
CI	confidence interval
TNM	Tumor–Node–Metastasis classification

## References

[1] Ginsberg R.J., Rubinstein L.V. (1995). Randomized trial of lobectomy versus limited resection for T1 N0 non-small cell lung cancer. Lung Cancer Study Group. Ann. Thorac. Surg..

[2] Saji H., Okada M., Tsuboi M., Nakajima R., Suzuki K., Aokage K., Aoki T., Okami J., Yoshino I., Ito H. (2022). Segmentectomy versus lobectomy in small-sized peripheral non-small-cell lung cancer (JCOG0802/WJOG4607L): A multicentre, open-label, phase 3, randomised, controlled, non-inferiority trial. Lancet.

[3] Altorki N., Wang X., Kozono D., Watt C., Landrenau R., Wigle D., Port J., Jones D.R., Conti M., Ashrafi A.S. (2023). Lobar or Sublobar Resection for Peripheral Stage IA Non-Small-Cell Lung Cancer. N. Engl. J. Med..

[4] Gross D.J., Hsieh M.S., Li Y., Dux J., Rekhtman N., Jones D.R., Travis W.D., Adusumilli P.S. (2021). Spread Through Air Spaces (STAS) in Non-Small Cell Lung Carcinoma: Evidence Supportive of an In Vivo Phenomenon. Am. J. Surg. Pathol..

[5] Darras M., Ojanguren A., Forster C., Zellweger M., Perentes J.Y., Krueger T., Gonzalez M. (2021). Short-term local control after VATS segmentectomy and lobectomy for solid NSCLC of less than 2 cm. Thorac. Cancer.

[6] Dixon L.K., Barber E., Cook A., Boudour J., Conibear J., West D., Navani N., Cromwell D.A. (2025). Sublobar resection or lobectomy for stage Ia non-small cell lung cancer: A systematic review and meta-analysis. BMJ Open Respir. Res..

[7] Hattori A., Suzuki K., Takamochi K., Wakabayashi M., Sekino Y., Tsutani Y., Nakajima R., Aokage K., Saji H., Tsuboi M. (2024). Segmentectomy versus lobectomy in small-sized peripheral non-small-cell lung cancer with radiologically pure-solid appearance in Japan (JCOG0802/WJOG4607L): A post-hoc supplemental analysis of a multicentre, open-label, phase 3 trial. Lancet Respir. Med..

[8] Brunelli A., Decaluwe H., Gonzalez M., Gossot D., Petersen R.H., Augustin F., Assouad J., Baste J.M., Batirel H., Falcoz P.E. (2023). European Society of Thoracic Surgeons expert consensus recommendations on technical standards of segmentectomy for primary lung cancer. Eur. J. Cardiothorac. Surg..

[9] Jacobs R.C., Rabin E.E., Logan C.D., Bharadwaj S.N., Yang H.C., Bell R.D., Cerier E.J., Kurihara C., Lung K.C., Avella Patino D.M. (2025). Pathologic upstaging and survival outcomes for patients undergoing segmentectomy versus lobectomy in clinical stage T1cN0M0 non-small cell lung cancer. JTCVS Open.

[10] Suzuki Y., Dhupar R., Sarkaria I.S., Christie I.G., Mazur S.N., Pennathur A., Luketich J.D., Levy R.M., Landreneau R.J., Schuchert M.J. (2025). Occult Node Detection With Lobectomy Versus Segmentectomy for Stage IA NSCLC. JTO Clin. Res. Rep..

[11] Ryuko T., Okazaki M., Mitsuhashi T., Suzawa K., Shien K., Ueno T., Fujiwara T., Watanabe M., Inokawa H., Misao T. (2025). Comparable Clinical Outcomes Between Segmentectomy and Lobectomy for NSCLC With Unsuspected N1/N2: A Multicenter Real-World Data Study. Ann. Thorac. Surg..

[12] Abdallat M., Leo R.T., Sugarbaker E.A., McAllister M., Xie Y., Mazzola E., Silvestri M., Bueno R., Jaklitsch M., Ugalde P. (2026). Segmentectomy vs Lobectomy for Occult N1 in Non-Small Cell Lung Cancer: Is Less More?. Ann. Thorac. Surg..

[13] Liou D.Z., Chan M., Bhandari P., Lui N.S., Backhus L.M., Shrager J.B., Berry M.F. (2022). Lobar versus sublobar resection in clinical stage IA primary lung cancer with occult N2 disease. Eur. J. Cardiothorac. Surg..

[14] Mynard N., Nasar A., Rahouma M., Lee B., Harrison S., Chow O., Villena-Vargas J., Altorki N., Port J. (2022). Extent of Resection Influences Survival in Early-Stage Lung Cancer With Occult Nodal Disease. Ann. Thorac. Surg..

[15] Razi S.S., Nguyen D., Villamizar N. (2020). Lobectomy does not confer survival advantage over segmentectomy for non-small cell lung cancer with unsuspected nodal disease. J. Thorac. Cardiovasc. Surg..

[16] Bongiolatti S., Mugnaini G., Salvicchi A., Gonfiotti A., Borgianni S., Viggiano D., Voltolini L. (2025). Completion lobectomy after thoracoscopic segmentectomy on the left side should be approached with thoracotomy. J. Thorac. Dis..

[17] Watanabe T., Fujino K., Sakane T., Handa Y., Doi T., Kawatani N., Nakazawa S., Iida T., Hanawa R., Shinohara S. (2025). Completion lobectomy following segmentectomy for malignant lung tumors: A multi-institutional study of surgical feasibility, oncologic outcomes, and diagnostic challenges (ESSG-02 study). Transl. Lung Cancer Res..

[18] Amir-Behghadami M., Janati A. (2020). Population, Intervention, Comparison, Outcomes and Study (PICOS) design as a framework to formulate eligibility criteria in systematic reviews. Emerg. Med. J..

[19] Luo X., Hayanga J.W.A., Mehaffey J.H., Lamb J., Campbell S., Reddy S., Badhwar V., Toker A. (2024). Clinical stage IA non-small cell lung cancer with occult pathologic N1 and N2 disease after segmentectomy: Does a completion lobectomy justify?. Eur. J. Cardiothorac. Surg..

[20] Nobel T.B., Tan K.S., Adusumilli P.S., Bains M.S., Downey R.J., Gray K., Huang J., Isbell J.M., Molena D., Park B.J. (2024). Outcomes of Patients Undergoing Segmentectomy for Occult Node-Positive Clinical Stage IA Lung Cancer. Ann. Thorac. Surg..

[21] Iwai Y., Tasoudis P., Agala C.B., Khoury A.L., O’Hara Garcia D.N., Long J.M. (2024). Lobectomy versus segmentectomy in patients with T1N2 non-small cell lung cancer: An analysis of the National Cancer Database. JTCVS Open.

[22] Lutfi W., Schuchert M.J., Dhupar R., Ekeke C., Sarkaria I.S., Christie N.A., Luketich J.D., Okusanya O.T. (2019). Node-Positive Segmentectomy for Non-Small-Cell Lung Cancer: Risk Factors and Outcomes. Clin. Lung Cancer.

[23] Stamatis G., Leschber G., Schwarz B., Brintrup D.L., Flossdorf S., Passlick B., Hecker E., Kugler C., Eichhorn M., Krbek T. (2022). Survival outcomes in a prospective randomized multicenter Phase III trial comparing patients undergoing anatomical segmentectomy versus standard lobectomy for non-small cell lung cancer up to 2 cm. Lung Cancer.

[24] Huang L., Brunelli A., Stefanou D., Zanfrini E., Donlagic A., Gonzalez M., Petersen R.H. (2025). Unforeseen nodal upstaging in patients undergoing segmentectomy without frozen section: A multicenter retrospective cohort study. Surg. Endosc..

[25] Nomori H., Mori T., Izumi Y., Kohno M., Yoshimoto K., Suzuki M. (2012). Is completion lobectomy merited for unanticipated nodal metastases after radical segmentectomy for cT1 N0 M0/pN1-2 non-small cell lung cancer?. J. Thorac. Cardiovasc. Surg..

[26] Huang L., Petersen R.H. (2024). Impact of number of dissected lymph nodes on recurrence and survival following thoracoscopic segmentectomy for clinical stage I non-small cell lung cancer. Lung Cancer.

[27] Adachi H., Ito H., Nagashima T., Isaka T., Murakami K., Shigefuku S., Kikunishi N., Shigeta N., Kudo Y., Miyata Y. (2025). Mediastinal lymph node dissection in segmentectomy for peripheral c-stage IA (</=2 cm) non-small-cell lung cancer. J. Thorac. Cardiovasc. Surg..

[28] Ding H., Wang H., Xu L., Song N., Jiang G. (2019). Survival and Resected Lymph Node Number During Sublobar Resection for N0 Non-Small Cell Lung Cancer 2 cm or Less. Ann. Thorac. Surg..

[29] Nagano M., Sato M. (2023). Impact of surgical margin after sublobar resection of lung cancer: A narrative review. J. Thorac. Dis..

[30] Huang L., Petersen R.H. (2025). Impact of Margin Distance on Locoregional Recurrence and Survival After Thoracoscopic Segmentectomy. Ann. Thorac. Surg..

[31] Nakagawa K., Watanabe S.I., Wakabayashi M., Yotsukura M., Mimae T., Hattori A., Miyoshi T., Isaka M., Endo M., Yoshioka H. (2025). Risk Factors for Locoregional Relapse After Segmentectomy: Supplementary Analysis of the JCOG0802/WJOG4607L Trial. J. Thorac. Oncol..

