# Supplementary material for: Nodal Upstaging and Oncologic Outcomes After Segmentectomy Versus Lobectomy for Early-Stage Non-Small Cell Lung Cancer
Source: Cancers (Basel). 2026 Mar 23;18(6):1039. doi: 10.3390/cancers18061039 (PMC13024766; doi:10.3390/cancers18061039)
Supplement: Supplementary file 1 [file cancers-18-01039-s001.zip › cancers-4205350-supplementary.pdf]

Supplementary Materials

Table S1. Quality Assessment of Included Studies Using the Newcastle–Ottawa Scale (NOS)

Study	Selection (0–4)	Comparability (0–2)	Outcome (0–3)	Total Score (/9)	Quality Level
Jacobs 2025	4	2	3	**9**	High
Suzuki 2025	3	1	3	**7**	Moderate
Ryuko 2025	3	2	3	**8**	High
Abdallat 2026	3	2	3	**8**	High
Razi 2020	4	2	3	**9**	High
Mynard 2022	4	2	3	**9**	High
Liou 2022	3	2	3	**8**	High
Luo 2024	4	2	3	**9**	High
Nobel 2024	3	1	3	**7**	Moderate
Iwai 2024	3	2	3	**8**	High
Lutfi 2019	3	1	3	**7**	Moderate
Huang 2025	3	1	3	**7**	Moderate

The Newcastle–Ottawa Scale (NOS) was used to assess the methodological quality of observational studies based on three domains: selection of study groups (maximum 4 stars), comparability of cohorts (maximum 2 stars), and outcome assessment (maximum 3 stars). Studies scoring 7–9 points were considered high quality, while those scoring 5–6 points were considered moderate quality.
